# Exploration of Secondary Metabolites in *Platostoma menthoides* (L.) Using Ethyl Acetate Extract and Its Antibacterial, Antioxidant, and Larvicidal Activities

**DOI:** 10.3390/toxics13010051

**Published:** 2025-01-11

**Authors:** Pavithra Senthilkumar, Subbu Thavamurugan, Aravinth Annamalai, Prabhu Kolandhasamy, Vasanthy Muthunarayanan, Nandhini Selvaraj, Lakshmiprabha Azhagiyamanavalan, Ramachandran Vinayagam

**Affiliations:** 1 Department of Botany, Bharathidasan University, Tiruchirappalli 620024, Tamilnadu, India; paviresearch22@gmail.com (P.S.); nizer934@gmail.com (S.T.); nandhiniresearch16@gmail.com (N.S.); 2 Department of Marine Science, Bharathidasan University, Tiruchirappalli 620024, Tamilnadu, India; amaravinth08@gmail.com (A.A.); drprabhu.marine@gmail.com (P.K.); 3 Department of Environmental Biotechnology, Bharathidasan University, Tiruchirappalli 620024, Tamilnadu, India; vasanthy@bdu.ac.in; 4 Department of Biotechnology, Institute of Biotechnology, School of Life and Applied Sciences, Yeungnam University, Gyeongsan 38541, Republic of Korea

**Keywords:** *Platostoma menthoides*, FT-IR, GC–MS, antibacterial, antioxidant, larvicidal activity

## Abstract

Recently, there has been a growing demand for plant-based products to treat a range of health conditions. *Platostoma menthoides* (L.), a member of the Lamiaceae family, is widely known for its versatile therapeutic properties. The primary aim of this study is to analyze and identify the secondary metabolites found in the extract of *P. menthoides* obtained using ethyl acetate and to assess its antioxidant, antimicrobial, and mosquito larvicidal properties for the first time. For the chemical profiling, a GC–MS analysis of the extract was conducted, and it showed the presence of various phytoconstituents, and the FT-IR spectrum revealed the functional groups associated with them. The quantitative phytochemical estimations revealed values of 34.87 ± 0.53 mg of GAE equivalents/g and 22.19 ± 1.11 mg of QUE equivalents/g, respectively, for total phenolic and flavonoid content. The biological studies of *P. menthoides* extract showed potent mosquito larvicidal activity against fourth instar larvae of *Aedes aegypti* at a 100 µg/mL concentration with LC_50_ and LC_90_ values of 81.328 and 161.471 µg/mL, respectively. Moreover, the DPPH and FRAP radical scavenging potentials were evaluated, and the results revealed their ability to scavenge the free radicals. The extract also showed higher antibacterial effects against gram-negative bacteria when compared to gram-positive bacteria. All these findings suggest that *P. menthoides* is a rich source of phytoconstituents with various medicinal applications and can be used as an antioxidant, antibacterial, and mosquito-larvicidal agent.

## 1. Introduction

Herbal medicines are getting profound attention all over the world based on their high efficiency and eco-friendly nature [1]. Approximately 80% of the global population relies on herbal medicine to treat various health conditions [2]. Different varieties of medicinal plants were previously reported for their versatile medicinal applications. They serve as an effective alternative to synthetic medicines because of their inexpensive and easy availability, along with fewer side effects at desirable dosages. To explore the medicinal significance of every drug, it becomes mandatory to perform the phytochemical screening. Such screening helps to establish the active ingredient and its standardization [3]. The specific biological activities of medicinal plants directly depend on the phytoconstituents present in them [4].

The mint family comprises many essential plants with versatile medicinal properties against various ailments. Various members of the family Lamiaceae have been reported for their extensive medicinal applications, such as antioxidant, anti-inflammatory, antimicrobial, anticancer, insecticidal, and antiviral properties [5]. Various important phytoconstituents, such as carvacrol, ursolic acid, eugenol, stearic acid, *β*-sitosterol, stigmasterol, oleanolic acid, rutin, chlorogenic acid, caryophyllene, limonene, bornyformate, and humulene [6], are reported from many Lamiaceae plants.

*Platostoma menthoides* (L.) *A.J. Paton (Ocimum menthoides Lin.* and *Geniosporum prostratum* [L.] *Benth.*) is an important unexplored family member of Lamiaceae, and it has extensive uses in various traditional medicines for the treatment of fever and the common cold in children as well as various other illnesses [7]. All such traditional uses pay special attention to this plant species, and scientific validation for its traditional claim is of great interest. As far as we are aware, there are only a few reports available describing the biological activities of *P. menthoides,* focusing on the antipyretic activity of the extract obtained using ethyl alcohol and the insecticidal activity of the precocene I compound extracted from the essential oil [8,9].

In this scenario, *P. menthoides* is considered an underexplored species despite its high medicinal values. In the future, this medicinal variety may serve as a reservoir for the development of various drug sources. In this context, the present study aims to investigate the phytochemical components and assess the biological activity spectrum of *P. menthoides.* This happens to be the first report for this highly potent unexplored medicinal plant. The present study comprises the qualitative and quantitative phytochemical profiling of ethyl acetate extract obtained from *P. menthoides* through colorimetric assays and FT-IR and GC–MS analyses. Further, a biological evaluation of the antioxidant, antibacterial and insecticidal properties of *P. menthoides* has been conducted and is also discussed in this paper.

## 2. Materials and Methods

### 2.1. Plant Collection and Identification

The *P. menthoides* sample was collected from the campus of Bharathidasan University in Tiruchirappalli. Dr. M.U. Sharief, Scientist and Head at the Botanical Survey of India, Coimbatore, authenticated the specimen (Voucher specimen no: BSI/SRC/5/23/22/Tech/369).

### 2.2. Preparation of Extract

Freshly gathered *P. menthoides samples* were thoroughly cleaned and rinsed with sterile distilled water to eliminate any dust and soil particles and then shade-dried. Once dried, the samples were ground into a powder using a blender and were stored in a container that was airtight until needed for experimentation. Subsequently, 25 g of the powdered sample was extracted with 500 mL of ethyl acetate using a Soxhlet apparatus. The extract was then concentrated with a rotary vacuum evaporator and was used for future assays. It was stored at 4 °C [10].

### 2.3. Phytochemical Studies

The ethyl acetate extract of *P. menthoides* was subjected to various qualitative phytochemical tests for the detection of alkaloids (Mayer’s test), flavonoids (aluminum-chloride test), steroids (Salkowski test), triterpenoids (Liebermann–Burchard reaction), reducing sugar (Fehling’s reagent), sugar (Fehling’s reagent), phenolic compounds (lead acetate test), glycosides (Keller–Killiani’s test), saponins (Foam test), tannins (FeCl_2_ test), anthraquinones (Modified Bontrager’s test), and aminoacids (Ninhydrin test) conferring to standard methods [11].

#### 2.3.1. Determination of Total Phenol Content

The total phenolic content was determined using the Folin–Ciocalteu method. The dried crude extract was dissolved in distilled water to achieve a concentration of 5 mg/L. The Folin–Ciocalteu reagent was prepared by diluting it five times with distilled water. Then 0.2 mL of the reagent was added to 1.6 mL of the diluted extract and vortexed for 3 min. Following this, 0.2 mL of 10% sodium carbonate was added, and the mixture was left to stand at room temperature for 30 min. After incubation, absorbance was measured at 765 nm using a spectrophotometer [12,13]. This procedure was performed three times consecutively, ensuring no errors occurred. Gallic acid (GAE) was used as a standard, and phenol was used in place of the stock solution to create the calibration curve. Using Equation (1) below, the total amount of phenol in the sample was estimated in milligrams of GAE per g of sample (mg GAE/g of the sample):

(1) $$GAE=\frac{C\times V}{M}$$

where *C* is the amount of *GAE* in mg/mL as determined by the 109-calibration curve; *V* is the volume of extract in milliliters; and *M* is the weight of the extract in grams.

#### 2.3.2. Determination of Total Flavonoid Content

The total flavonoid content of the samples was estimated using the aluminum chloride colorimetric method, as described [14]. To begin, 1 mL of the extract was mixed with 5 mL of deionized water, followed by the addition of 3 mL of 5% sodium nitrate (NaNO_2_). Next, 0.3 mL of 10% aluminum chloride (AlCl_3_) was added to the content, with both NaNO_2_ and AlCl_3_ prepared using distilled water. After 6 min, 2 mL of 1 M sodium hydroxide was added, and distilled water was used to bring the volume to 10 mL. The mixture was then incubated in the dark for about 30 min. The OD was measured at 510 nm using a spectrophotometer, and the total flavonoid content was calculated using the following Equation (2):

(2) $$QE=\frac{C\times V}{M}$$

where *C* is the quercetin concentration determined from the calibration curve in mg/mL, *V* = amount of diluted sample in mL, and *M* is the weight of the extract in grams.

### 2.4. FT-IR Analysis

The FT-IR analysis of the plant extract powder revealed that it contains different secondary metabolites and other biologically active substances. The Fourier transform infrared spectrometer (FT-IR) is possibly the most accurate means of determining the types of chemical connections and functional groupings found in plants. An FT-IR analysis was performed for the plant extract of *P. menthoides*. The extract powder was placed into an FT-IR spectroscope (Shimadzu, Tokyo, Japan), which had a scanning frequency range of about 400 to 4000 cm^−1^ and an accuracy of 4 cm^−1^ [15].

### 2.5. GC–MS Analysis (Gas Chromatography–Mass Spectrometry)

A phytochemical investigation of ethyl acetate extract of *P. menthoides* was conducted using GC–MS analysis. An AOC-20s auto-sampler, an AOC-20i auto-injector, and gas chromatography (GC-2010) interfaced with a mass spectrometer composed the GC–MS apparatus (GC-MS Agilent 8890, Wilmington, DE, USA). The following were the GC–MS system testing requirements: an SHRxi-5sil-MS capillary standard non-polar column made of dimethylpolysiloxane (dimensions: 30.0 m, diameter: 0.25 mm, 139 film thickness: 0.25 m). It was performed using an electron ionization potential system that contained ionization energy of 70 eV. A continuous flow rate of 1.20 mL/min and an injector volume were used for the gas that served as the carrier, which was 99.99% helium gas (5 μL) (spilt ratio: 15:1). The injector’s range was 250 °C, whereas the source of ion conditions was 200 °C. For two minutes, the oven’s temperature was set at 50 °C isothermal; after that, it was raised to 280 °C for 10 min. The scan range for the mass spectra was 50–500 *m*/*z*, with a scanning period of 0.3 s, and they were recorded at 70 eV. The GC was operational for a total of 40 min. We determine the proportion by comparing the average peak area of each component with respect to the total area, allowing us to estimate the approximate amount of each component. By employing GC–MS real-time analytical Open Lab-CDS, version: 2.5 software, the obtained chromatograms and spectra were analyzed [16].

### 2.6. Estimation of Antioxidant Activity

#### 2.6.1. 2,2-Diphenyl-1-Picrylhydrazyl (DPPH) Radical Scavenging Assay

The DPPH radical scavenging activity of different concentrations of the ethyl acetate extract of *P. menthoides* was assessed using the standard DPPH assay method [17]. Briefly, 0.1 mL of the crude ethyl acetate extract at concentrations ranging from 20 to 100 µg/mL was mixed with 3 mL of DPPH and incubated at room temperature for 30 min. The absorbance was then evaluated using a UV–vis spectrophotometer at 517 nm. The IC_50_ value of ethyl acetate extract was noted [18].

#### 2.6.2. Ferric Reducing Antioxidant Potential Assay (FRAP)

The methodology was used to assess the antioxidant potential of ethyl acetate extract prepared from various concentrations to reduce ferric ion (Fe^3+^) to ferrous ion (Fe^2+^). The freshly prepared reactive mixture was made by combining 20 mM ferric chloride (FeCl_3_), 10 mM 2,4,6-tris(2-pyridyl)-s-triazine (TPTZ), and 300 mM acetate buffer (pH 3.6) in a 10:1:1 combination. On the day of analysis, the TPTZ reagent was formulated. The same concentration of ascorbic acid standards was used to measure the values [19]. Then, 20–100 µg/mL of samples were placed in the 96-well plate. The test samples were incubated in a microplate for about 30 min at 37 °C. Then the frequency of 593 nm absorbance was observed. Each analysis was performed three times. FRAP values were compared as mg ascorbic acid equivalents per liter [20].

### 2.7. Anti-Bacterial Activity

#### Agar Disc Diffusion Assay

The test bacterial cultures *Staphylococcus phyogens* (MTCC-96), *Salmonella typhi* (MTCC-3858), *Enterococcus facelis* (MTCC-439), and *Pseudomonas aeruginosa* (MTCC-1688) were purchased from the MTCC. The antibacterial activity of the ethyl acetate extract of *P. menthoides* was assessed using the disc diffusion method. Bacteria-nutrient media was made, and the mixture was placed in sterile Petri dishes. Approximately 0.2 mL of the standard bacterial inoculum was applied to the agar medium using a sterile cotton swab. On the inoculated agar medium, the discs impregnated with plant extract were inserted. The 10 μg/disc of ampicillin was used as a standard to evaluate the sensitivity of each microbial species. Each plate was subsequently incubated for 24 h at 37 °C. After the incubation period, the inhibition zone around the disks was measured [15].

### 2.8. Mosquito-Larvicidal Activity

#### 2.8.1. Mosquito Larvae and Egg Culture

The eggs and larvae of the mosquito (*Aedes aegypti*) were obtained from the Vector Control Research Centre (ICMR), Madurai (India). They were transferred to 25 × 25 × 20 cm trays that were filled with adequate water and maintained in laboratory conditions (a temperature of 28 ± 2 °C and a 12:12 L/D photoperiod). The young ones were fed with dog biscuits + yeast on a routine basis in the ratio of 3:1 until they reached the fourth instar stage [21].

#### 2.8.2. Larvicidal Bioassay

A bioassay to understand the larvicidal activity was conducted as per the WHO protocol (1996) with minor modifications. Twenty-five healthy fourth instar larvae were placed in separate plastic cups containing 200 mL of tap water. A control group was set up with just tap water. Different concentrations of ethyl acetate extract (20, 40, 60, 80, and 100 µg/mL) were added to the individual chambers. Following 24 h of treatment, the dead larvae number was recorded. The experiments were performed in triplicate, and the larval mortality percentage was calculated using Abbott’s formula, as shown in Equation (3) [22].

(3) $$Mortaliy\;(\%)=\frac{Survival\;in\;the\;untreated\;control-Survival\;in\;the\;treated\;sample}{Total\;no.\;of\;Control\;larvae}\times 100$$

#### 2.8.3. Analysis of Histopathology

For the histopathological examination of the mosquito larvae, 24 h after treatment (and control), *Aedes aegypti* larvae were fixed in diluted formaldehyde for 2 h at 5 °C. The formed block temperature was maintained at 25 °C for up to 5 h, and the blocks were sectioned into 8 μm thick ribbons using a microtome. The gut sections were stained with haematoxylin and eosin. After air-drying, the slides were examined and photographed under an LED-light microscope (Labomed—LX-300_LED, Los Angeles, CA, USA) at 400× magnification [23].

#### 2.8.4. Statistical Analysis

All numerical analyses were conducted in triplicate. The data obtained from the toxicity assay were subjected to Probit analysis, from which various lethal concentrations were determined. SPSS version 16 was used for variance analysis ANOVA at *p* < 0.05 and other statistical analyses.

## 3. Results and Discussion

### 3.1. Phytochemical Studies Analysis

The phytochemical screening of crude extract (*P. menthoides)* gives insight into the presence of various active phytoconstituents that are responsible for the therapeutic potentials [24]. The extract prepared using ethyl acetate *P. menthoides* reveals the presence of various classes of phytoconstituents such as steroids, alkaloids, anthraquinone glycosides, flavonoids, tannins, phenols, carbohydrates, saponins, triterpenoids, and citric acid (Table 1). The *P. menthoides* extract’s phenolic and flavonoid concentration is compatible with studies that show its involvement in lowering oxidative stress in cells, potentially preventing chronic conditions like cancer and heart diseases [25]. The presence of these major classes of phytoconstituents implies the richness of bioactive molecules present in the crude extract, and this could be the reason behind their effective therapeutic potential.

For the quantitative estimations of total phenolic and flavonoid content in the ethyl acetate extract of *P. menthoides*, Folin–Ciocalteu reagent and aluminum chloride photometry methods were attributed. The results of the quantitative estimation revealed the presence of 231.28 ± 0.02 mg/g GAE of the phenolics, whereas 196.84 ± 0.87 mg/g QUE of flavonoids was recorded in the extract. Consequently, the total phenolic and flavonoid estimations are depicted in Table 2. Contingent on the obtained results, it is revealed that *P. menthoides* extract prepared using ethyl acetate has phenolic and flavonoid contents, and it makes this species an important chemical variety with multiple biological actions. Based on the available reports, phenolic compounds can exhibit antioxidant properties by acting as reducing agents and quenching free radicals [26]. It is also found to be responsible for various biological activities such as cytotoxic activity, antioxidant activity, and antihepatotoxic activities. Meanwhile, the flavonoids are also reported for their various medicinal applications, such as antimicrobial, antioxidant, anti-inflammatory, and hypoglycemic properties [27,28,29,30,31,32,33]. All these findings of the phytochemical screening of *P. menthoides* reveal its unique properties as a rich source of medicinal agents.

### 3.2. FTIR Analysis

**Figure 1:**
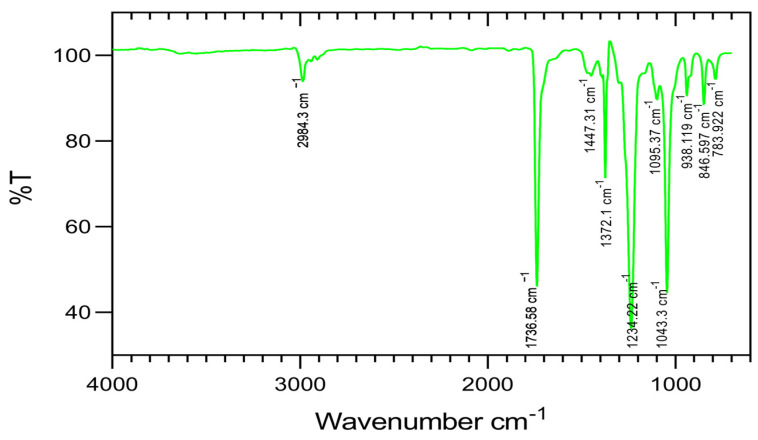
Functional group identified from FT-IR spectrum ethyl acetate extract of *P. menthoides*.

For the identification of various functional groups present in the extract, an FT-IR profiling was conducted (Figure 1). It can provide a rapid and non-destructive investigation of plant extract for the identification of phytoconstituents. Various constituents present in the plant extract consist of different bonds (C-H, C=O, C-H, O-H, C-N, C=O, CO-O-CO, O-H, C-Cl, and C–Cl). These bonds can be easily identified by analyzing the distinct frequency absorption band in the infrared spectrum. The FT-IR spectra obtained for the *P. menthoides* ethyl acetate extract showed the presence of 3 major and 7 minor peaks (Table 3). A characteristic peak observed at 2984.3 cm^−1^ represents the C-H stretching medium vibrations of alkane. The broad and intense absorption band appeared at 1736.58 cm^−1^, indicating C=O stretching strong vibrations of esters; 1447.31 cm^−1^ indicates the presence of C-C stretching medium vibration of aromatics. The band at 1372.1 cm^−1^ corresponds to O-H bending medium vibrations of phenol. A band at 1234.22 cm^−1^ corresponds to C-N stretching with the functional group of aromatic amines; 1095.37 cm^−1^ indicates the C-N stretching corresponding to aliphatic amine; and 1043.3 cm^−1^ indicates CO-O-CO stretching vibrations of anhydride. The peak at the 938.199 cm^−1^ region is characteristic of an O-H bend with the carboxylic acids group. The peak at 846.597 cm^−1^ corresponds to the vibration of C-Cl stretching medium vibrations of alkyl halides, and 783.922 cm^−1^ indicates C-Cl stretching with the functional group of alkyl halides, respectively. Previously, an FT-IR profiling of Lamiaceae members reported the presence of alcohols, phenols, alkanes, alkynes, alkyl halides, aldehydes, carboxylic acids, and aromatic compounds [34,35]. The presence of aldehyde groups, as identified in the FT-IR analysis, is often associated with antibacterial properties due to their ability to disrupt microbial cell membranes [36]. This likely accounts for the potent antibacterial effect observed against both gram-positive and gram-negative bacteria. The obtained spectrum serves as a product’s fingerprint and reveals the various organic groups and molecular connections present in the investigated extract as different peaks or bands.

### 3.3. GC–MS Analysis

**Figure 2:**
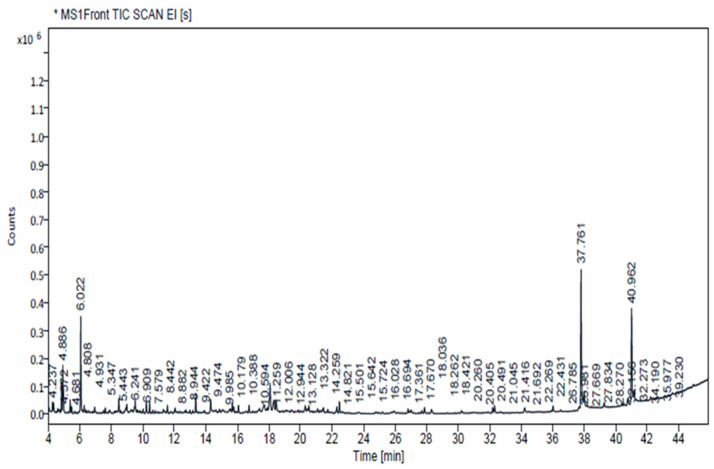

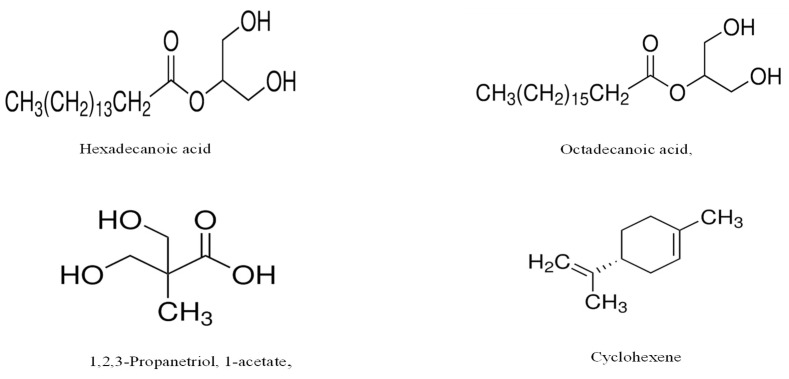
GC–MS chromatogram analysis of ethyl acetate extract of *P*. *menthoides.* The chemical structure of the four abundant compounds identified in the NIST library.

For the prior identification of phytoconstituents present in the *P. menthoides* extract, GC–MS profiling was carried out and the obtained output is depicted in Table S1. In total, 50 components were identified in the GC–MS chromatogram of ethyl acetate extract of *P. menthoides*. Compounds such as hexadecanoic acid, 2-hydroxy-1-(hydroxymethyl) ethyl ester, Octadecanoic acid, 2-hydroxy-1-(hydroxymethyl) ethyl ester, 1,2,3-Propanetriol, 1-acetate, cyclohexene, and 1-methyl-5-(1-methylethenyl)-, (R) are identified as more prominent compounds in the crude extract of *P. menthoides*. Details including the biological activities of major compounds obtained in the chromatogram are depicted in Figure 2. According to the results obtained, most of the identified components were identified as having pharmaceutical value. Hexadecenoic acid, 2-hydroxy-1-(hydroxymethyl) ethyl ester, also known as 2-palmitoylglycerol, was identified in the chromatogram at a retention time of 37.761 min with a probability of 55.79%. This compound exhibits notable anti-inflammatory, antioxidant, and anthelmintic properties [37]. Octadecanoic acid, 2-hydroxy-1-(hydroxymethyl) ethyl ester, commonly referred to as 2-stearoylglycerol, was detected in the chromatogram with a retention time of 40.962 min and a probability of 71.42%; 1,2,3-propanetriol, 1-acetate was detected in the chromatogram at a retention time of 6.022 min, with a probability of 78.19%. This compound exhibits significant antifungal, anticancer, and anti-inflammatory potential [38]. Cyclohexene, 1-methyl-5-(1-methylethenyl)-, (R), also called R)-(+)-Limonene, was identified in the chromatogram at a retention time of 4.886 min, with a probability of 19.27%. It demonstrated strong antimicrobial properties. Therefore, most of the components identified in the ethyl acetate extract are recognized for their antioxidant, antimicrobial, anti-inflammatory, anticancer, diuretic, and insecticidal activities [39].

### 3.4. Biological Studies

#### 3.4.1. Antioxidant Activity

**Figure 3:**
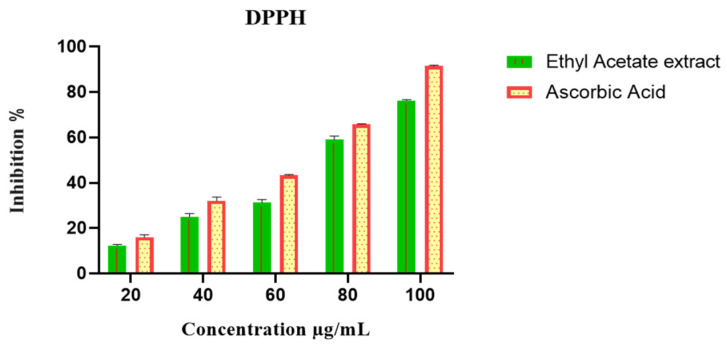
DPPH radical scavenging activity of ethyl acetate extract of *P. menthoides*.

**Figure 4:**
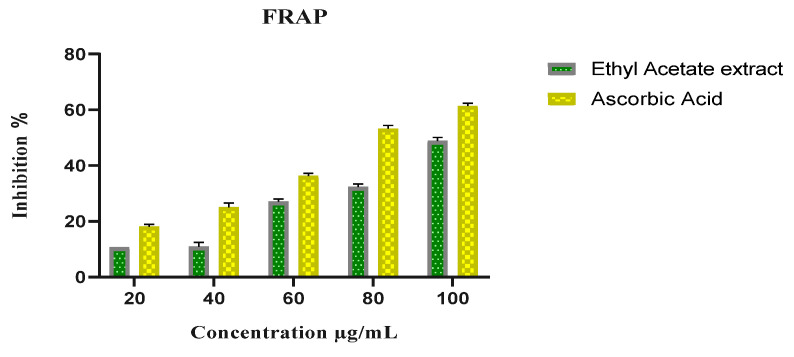
FRAP radical scavenging activity of ethyl acetate extract of *P. menthoides*.

The antioxidant potential of *P. menthoides* was investigated by using standard procedures such as DPPH and FRAP. The scavenging effect of plant extract on the DPPH and peroxide free radical was inhibited performed using ascorbic acid as the standard. The scavenging capability extract prepared using ethyl acetate at concentrations of 20, 40, 60, 80, and 100 μg/mL was compared to ascorbic acid, although the IC_50_ value of 60 μg/mL concentration resulted in antioxidant activity of 76.32 ± 0.34 (DPPH) and 48.85 ± 1.22 (FRAP) (Figure 3 and Figure 4). Earlier studies observed antioxidant properties of *Platostoma africanum* using phenolic constituent synergistic activity [40,41]. From the results of the DPPH and FRAP assays, the presence of gallic acid and ascorbic acid, which were extracted from the plant extracts, may be primarily responsible for the antioxidant capacity of *P. menthoides.* This study strongly indicates that a single approach to determining antioxidant capacity cannot reveal the complex antioxidant potential of a natural compound.

#### 3.4.2. Antibacterial Activity

**Figure 5:**
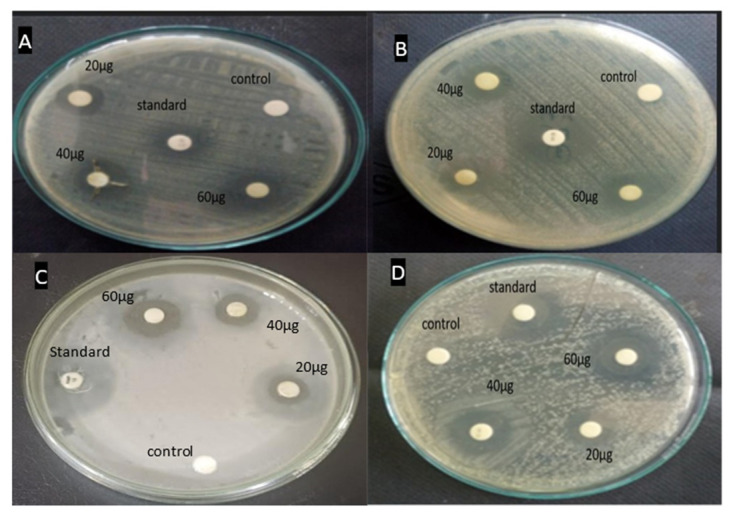
Antibacterial analysis by disc diffusion assay of ethyl acetate extract of *P. menthoides*; (**A**)-*Streptococcus pyogenes*, (**B**)-*Enterococcus faecalis*, (**C**)-*Pseudomonas aeruginosa*, and (**D**)-*Salmonella typhi*.

The antibacterial performance of the ethyl acetate extracts of *P. menthoides* was performed against four bacterial strains. The results showed that the ethyl acetate extract considerably inhibits both gram-positive and gram-negative bacterial strains. Photographs of the results of the disk diffusion assay are included in Figure 5. The antibacterial activity of the ethyl acetate extract was similar against both gram-positive and gram-negative bacteria (Table 4). The results of ethyl acetate plant extract showed the highest inhibitory activity that was recorded against *Pseudomonas aeruginosa* (18 ± 0.22 mm); the minimum inhibitory effect was noted in *Enterococcus faecalis* (15 ± 0.62), followed by *Salmonella typhi* (14 ± 1.12) and *Streptococcus pyogenes* (12 ± 0.67). As expected, zero inhibition was observed in the control. Ngezahayo et al. [42] reported that the oil prepared using *P. hispidium* demonstrated antibacterial activity against all six tested bacterial strains, but *S. saprophyticus* and *S. flexneri* exhibited considerably stronger antibacterial activity. Some other studies examined the antioxidant and antibacterial properties of 12 varieties of the different Lamiaceae families such as *Mentha, Salvia,* and *Siderites*, which were gathered in Greece and obtained using polyphenols. These actions were also associated with the extract of polyphenolic content [43]. Previous studies by ERSÖZ et al. [44] have demonstrated that *Lamium geranium* L. subsp. *laevigatum* and *Lamium garganicum* L. subsp. *pulchrum* possess antibacterial properties. Literature data indicate that numerous species within the Lamiaceae family produce various metabolites with significant antibacterial effects. These metabolites hold potential for the development of new antibacterial drug discovery.

Our study confirmed this assertion of the antibacterial activity of *P. menthoides* extracts. The ethyl acetate extract contains a considerable variety of phytochemicals, including alkaloids, flavonoids, phenols, quinones, saponins, tannins, steroids, and terpenoids, which inhibit the growth of pathogenic bacterial species. Many commercially available bioactive compounds are typically sourced from these phytochemicals, which are known for their antimicrobial properties.

#### 3.4.3. Larvicidal Activity

**Figure 6:**
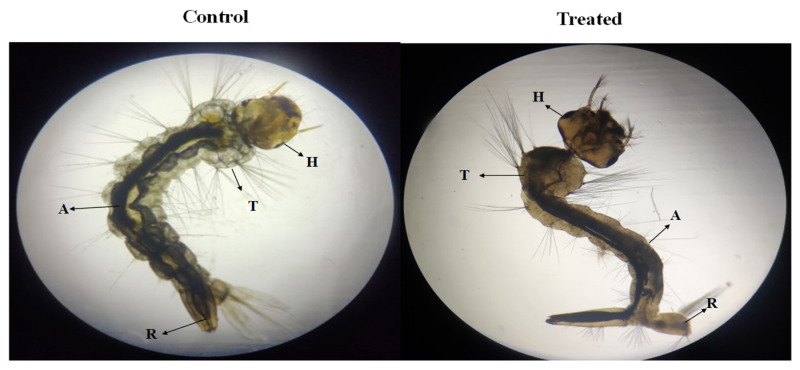
Mosquito larvicidal activity of *P. menthoides* against *Ae. Aegypti.* Control and treated mosquito larvae *Aedes aegypti*: No deformities were witnessed in the control group of larvae. The malformation was observed in the bodies of the larvae with ethyl acetate extract from *P. menthoides*. H—Head; T—Throax; A—Abdomen; R—Respiratory siphon.

The larvicidal effects of the crude ethyl acetate extract against the third instar larvae of *Aedes aegypti* are represented in Figure 6 and Table 5. The probit regression responses of the ethyl acetate extract tested revealed that the larvicidal activities after 24 h ranged from the LC_50_ of 81.328 µg/mL to the LC_90_ of 161.471 µg/mL. Previously, *Mentha piperita* L. leaves (LC_50_ = 0.42 g/mL) and *Piper betle* L. leaves (LC_50_ = 0.69 g/mL) were both shown to take effective larvicidal action against *Aedes aegypti* [45,46]. The milky sap of *Ficus carica* is poisonous to early fourth-stage *Aedes aegypti* larvae, with an LC_50_ value of 10.2 µg/mL and an LC_90_ value of 42.3 µg/mL [47]. The present study has demonstrated that *P. menthoides* ethyl acetate extract has larvicidal effects on *Aedes aegypti*. This ethyl acetate extract of *P. menthoides* showed promising larvicidal activity against disease-carrying mosquitoes *A. aegypti,* while the control group did not show any deformity of mosquito larva.

#### 3.4.4. Histological Analysis

**Figure 7:**
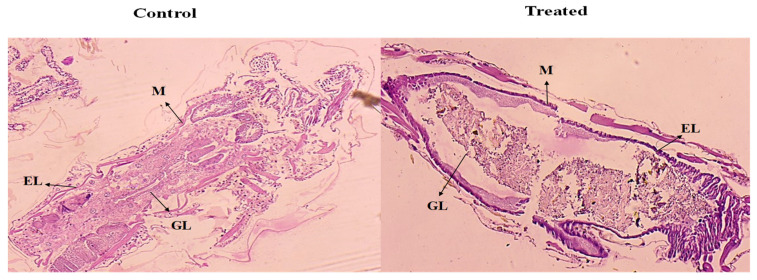
Histopathological evaluation of the control and treated larvae *Aedes aegypti*. Blocked arrows showed various cellular regions. M—Muscles; EL—Epithelium layer; GL—Gut lumen.

The maximum mortality rate of *A. aegypti* larvae was observed during the 24 h exposure period via a histological examination, represented in Figure 7. The histological analysis of ethyl acetate extract of *P. menthoides* showed promising histological changes in mosquito larva such as disorganized and damaged epithelial cell layers, as well as impacted midgut, gut lumen, and muscles, but in the control group, mosquito larvae did not show any deformity in the histological observation. Similarly, a previous study conducted by Moola et al. [48] reported the *P. menthoides* ethyl acetate-treated larvae had disorganized and damaged epithelial cell layers, as well as significantly impacted midgut, gut lumen, and muscles, according to histology.

## 4. Conclusions

The crude ethyl acetate extract of *P. menthoides* contained a substantial amount of pharmaceutically valuable phytochemicals. Additionally, the extract, enriched with these phytochemicals, exhibited considerable antibacterial activity against four bacterial pathogens. The DPPH and FRAP analyses confirmed the extract’s significant antioxidant properties. A GC–MS analysis identified approximately 10 major pharmaceutically valuable components in this ethyl acetate extract. Given the identified chemicals’ cytotoxic and antioxidant capabilities, this extract shows promise for future drug development, notably in cancer treatments and the treatment of oxidative stress-related disorders. The toxicity of ethyl acetate extract of *P. menthoides* suggests that it could be used along with the mosquito predators in the integrated vector control programs and promises a natural option for mosquito control, providing a safer and more sustainable solution than chemical pesticides for human subjects. Therefore, this is the first report regarding the larvicidal activity of *P. menthoides* leaf extract, and further research is needed to identify the specific larvicidal compounds in the extract. However, the subsequent studies should focus on isolating the specific bioactive chemicals responsible for the reported actions and conducting in vivo investigations to better understand their potential applications in human health and disease prevention.

## Figures and Tables

**Table 1:** Phytochemical constituents from *P. menthoides* ethyl acetate extract.

Phytochemicals Tested	Reagent Used	Ethyl Acetate
Steroids	Salkowski test	+
Triterpenoids	Liebermann–Burchard reaction	+
Reducing sugar	Fehling’s Reagent	++
Sugar	Fehling’s Reagent	+
Alkaloids	Mayers, Hagers test	+
Phenolic compounds	Lead acetate test	+++
Glycosides	Keller–Killiani’s test	-
Flavonoids	Aluminium Chloride test	+++
Saponins	Foam test	+
Tannins	FeCl_2_ test	-
Anthraquinones	Modified borntragers test	+
Aminoacids	Ninhydrin test	-

Note: The symbols refer to +++ appreciable, ++ moderate, + trace amounts, and - absence of secondary metabolites, respectively, in the ethyl acetate extracts.

**Table 2:** Total phenolic and flavonoid contents in *P. menthoides* ethyl acetate extract.

Concentrations (µg/mL)	Phenolic Content	Flavonoid Content
20	150.05 ± 0.01 ^e^	143.48 ± 0.32 ^e^
40	170.47 ± 0.01 ^d^	156.98 ± 0.57 ^d^
60	196.23 ± 0.05 ^c^	172.38 ± 0.86 ^c^
80	213.62 ± 0.04 ^b^	183.71 ± 0.25 ^b^
100	231.28 ± 0.02 ^a^	196.84 ± 0.87 ^a^

Data expressed as mean ± SD of three replicates; means bearing different superscripts ^(a,b,c,d,e)^ indicates a significant difference when compared with control; *p* < 0.05.

**Table 3:** Functional groups identified in FT-IR spectrum of *P. menthoides* ethyl acetate extract.

S. No	Frequency cm^−1^	Bond	Functional Group	Vibration/Intensity
1.	2984.3	C-H stretching	alkane	medium
2.	1736.58	C=O stretching	esters	strong
3.	1447.31	C-C stretching	aromatics	medium
4.	1372.1	O-H bending	phenol	medium
5.	1234.22	C-N stretching	aromatic amine	medium
6.	1095.37	C-N stretching	aliphatic amine	strong
7.	1043.3	CO-O-CO stretching	anhydride	strong, broad
8.	938.199	O-H bend	carboxylic acids	medium
9.	846.597	C-Cl stretching	alkyl halides	medium
10.	783.922	C-Cl stretching	alkyl halides	medium

**Table 4:** Antibacterial activity of *P. menthoides* ethyl acetate extract.

Bacteria Strains	20 µg	40 µg	60 µg	Ampicillin
*Streptococcus pyogenes*	5.4 ± 0.26	9.3 ± 0.01	12 ± 0.67	15 ± 1.11
*Enterococcus faecalis*	9.2 ± 0.12	13 ± 0.32	15 ± 0.62	14 ± 0.21
*Pseudomonas aeruginosa*	5.8 ± 0.12	10 ± 0.45	18 ± 0.22	21 ± 1.12
*Salmonella typhi*	10 ± 1.26	11 ± 0.32	14 ± 1.12	17 ± 1.20

**Table 5:** Mosquito-Larvicidal activities of *P. menthoides* ethyl acetate extract against *Ae. aegypti*.

Mosquito Vector	Sample	Con. (µg/mL)	LC50 µg/mL (LCL-UCL)	LC90 µg/mL (LCL-UCL)	χ2
*Ae. aegypti*	Ethyl acetate extract	20 40 60 80 100	81.32 (73.53–91.89)	161.47 (139.86–197.88)	3.33

## Data Availability

The data that support the findings of the study are available from the corresponding author upon responsible request.

## Supplementary Materials

The following supporting information can be downloaded at https://www.mdpi.com/article/10.3390/toxics13010051/s1: Table S1: GC MS profiling of *P. menthoides* ethyl acetate extract.
