# Supplementary material for: Exploration of Secondary Metabolites in Platostoma menthoides (L.) Using Ethyl Acetate Extract and Its Antibacterial, Antioxidant, and Larvicidal Activities
Source: Toxics. 2025 Jan 11;13(1):51. doi: 10.3390/toxics13010051 (PMC11769029; doi:10.3390/toxics13010051)
Supplement: Supplementary file 1 [file toxics-13-00051-s001.zip › toxics-3367601-supplementary.pdf]

Article

Exploration of secondary metabolites in *Platostoma menthoides* (L.) using ethyl acetate extract and its antibacterial, antioxidant, and larvicidal activities

Pavithra Senthilkumar[1], Subbu Thavamurugan[1], Aravinth Annamalai[2], Prabhu Kolandhasamy[2], Vasanthy Muthunarayanan[3], Nandhini Selvaraj[1], Lakshmiprabha Azhagiyamanavalan[1]* and Ramachandran Vinayagam[4]*

[1]Department of Botany, Bharathidasan University, Tiruchirappalli – 620 024, Tamil Nadu, India.

[2]Department of Marine Science, Bharathidasan University, Tiruchirappalli – 620024, Tamil Nadu, India

[3]Department of Environmental Biotechnology, Bharathidasan University, Tiruchirappalli – 620024, Tamil Nadu, India.

[4]Department of Biotechnology, Institute of Biotechnology, School of Life and Applied Sciences, Yeungnam University, Gyeongsan 38541, Korea.

*Correspondence: dralprabha@yahoo.com (L.A.A); rambio85@gmail.com (R.V)

Table S1. GC MS profiling of *P. menthoides* ethyl acetate extract.

S. No	R. T	Area of %	CAS	Molecular Weight	Molecular Formula	Compound Name
1.	3.757	16.24	56-81-5	92.09	$C_3H_8O_3$	Glycerin
2.	4.237	1.38	1225387-53-0	*83.09*	$C_3H_5N_3$	1H-Pyrazol-3-amine
3.	4.572	0.36	508-32-7	*136.23*	C_{10} H_{16}	Tricyclo[2.2.1.0(2,6)]heptane, 1,7,7-trimethyl
4.	4.681	0.43	35124-13-1	206.32	$C_{23}H_{22}O$	Irone α a
5.	4.808	1.82	527-84-4	134.21	$C_{10}H_{14}$	o-Cymene
6.	4.886	2.93	1461-27-4	136.23	C_{10} H_{16}	Cyclohexene, 1-methyl-5-(1-methylethenyl)-, (R)-
7.	4.931	1.21	100-51-6	108.14	$C_6H_5CH_2OH$	Benzyl alcohol
8.	5.347	1.00	62108-27-4	184.36	$C_{13}H_{28}$	Decane, 2,4,6-trimethyl
9.	5.443	0.64	105-90-8	*210.31*	$C_{13}H_{22}O_2$	-3,7-Dimethyl-2,6-octadien-1-yl propionate
10.	6.022	7.29	106-61-6	134.13	$C_5H_{10}O_4$	1,2,3-Propanetriol, 1-acetate
11.	6.241	0.58	62108-27-4	184.36	$C_{13}H_{28}$	Decane, 2,4,6-trimethyl
12.	7.579	0.43	17865-32-6	188.34	$C_9H_{20}O_2Si$	Silane, cyclohexyldimethoxymethy
13.	8.442	1.21	112-40-3	170.34	$C_{12}H_{26}$	Dodecane
14.	8.882	0.64	4748-78-1	134.17	$C_9H_{10}O$	Benzaldehyde, 4-ethyl
15.	8.944	0.84	122-99-6	138.16	$C_8H_{10}O_2$	Ethanol, 2-phenoxy
16.	9.422	0.39	5057-99-8	102.13	$C_5H_{10}O_2$	1,2-Cyclopentanediol, trans
17.	9.474	1.01	56618-58-7	202.29	$C_{11}H_{22}O_3$	Decanoic acid, 3-hydroxy-, methyl ester
18.	9.985	0.39	19790-87-5	*230.34*	$C_{13}H_{26}O_3$	Methoxyacetic acid, 6-ethyl-3-octyl ester
19.	10.179	1.01	106-26-3	152.24	$C_{10}H_{16}O$	Neral
20.	10.388	0.97	31295-56-4	212.41	$C_{15}H_{32}$	Dodecane, 2,6,11-trimethyl
21.	10.594	0.28	10147-41-8	353.00	$C_{18}H_{37}C_{lO}2S$	1-Octadecanesulphonyl chloride
22.	11.259	0.49	621-58-9	150.17	$C_9H_{10}O_2$	Phenol, 5-ethenyl-2-methoxy
23.	12.006	0.48	488-38-0	212.20	$C_7H_{16}O_7$	D-glycero-D-manno-Heptitol
24.	12.944	0.28	41370-29-0	*154.24*	$C_{10}H_{18}O$	Ethanol, 2-(3,3-dimethylcyclohexylidene
25.	13.128	0.28	408-14-0	*78.04*	$C_{14}H_{27}FO_2$	Fluoroacetic acid, dodecyl ester
26.	13.322	1.47	629-59-4	198.39	$C_{14}H_{30}$	Tetradecane
27.	14.259	1.57	61387-11-9	136.15	$C_8H_8O_2$	2,3-Trimethylene-4-pyrone
28.	14.821	0.34	959246-45-8	*272.42*	$C_{16}H_{32}O_3$	Methoxyacetic acid, 4-tridecyl ester
29.	15.501	0.42	192823-15-7	198.39	$C_{14}H_{30}$	Decane, 2,3,5,8-tetramethyl
30.	15.642	0.74	73105-67-6	296.236	$C_{12}H_{25}I$	1-Iodo-2-methylundecane
31.	16.028	0.76	128-39-2	*206.32*	$C_{14}H_{22}O$	Phenol, 2,6-bis(1,1-dimethylethyl)
32.	16.694	0.85	14905-56-7	240.5	$C_{17}H_{36}$	Tetradecane, 2,6,10-trimethyl
33.	17.361	0.68	-	344.7	$C_{19}H_{44}OSi_2$	6-Dimethyl(trimethylsilyl)silyloxytetradecane
34.	17.670	1.76	-	318.32	$C_{14}H_{22}O_8$	1,4-Diacetyl-3-acetoxymethyl-2,5-methylene-l-rhamnitol
35.	18.262	2.44	19467-01-7	208.21	$C_8H_{16}O_6$	Ethyl α-d-glucopyranoside
36.	20.405	0.43	-	286.4	$C_{17}H_{34}O_3$	Methoxyacetic acid, 2-tetradecyl ester
37.	20.491	0.59	10544-96-4	268.52	$C_{19}H_{40}$	Octadecane, 6-methyl
38.	21.045	0.35	458-35-5	180.20	$C_{10}H_{12}O_3$	4-((1E)-3-Hydroxy-1-propenyl)-2-methoxy phenol
39.	21.692	0.25	120-51-4	212.25	$C_{14}H_{12}O_2$	Benzyl Benzoate
40.	22.269	0.84	95008-11-0	294.6	$C_{21}H_{42}$	10-Heneicosene (c,t)
41.	22.431	1.11	593-45-3	254.49	$C_{18}H_{38}$	Octadecane
42.	26.785	0.45	57-10-3	256.4	$C_{16}H_{32}O_2$	n-Hexadecanoic acid
43.	26.981	0.66	1560-78-7	352.7	$C_{25}H_{52}$	2-Methyltetracosane
44.	27.669	0.31	83005-02-1	*325.31*	$C_{16}H_{30}C_{l2}O_2$	Dichloroacetic acid, tetradecyl ester
45.	28.270	0.67	10544-50-0	*256.5*	S_8	Cyclic octaatomic sulfur
46.	32.156	0.60	74685-33-9	280.5	$C_{20}H_{40}$	3-Eicosene, (E)
47.	35.977	0.74	55333-99-8	366.7	$C_{26}H_{54}$	Eicosane, 7-hexyl

48.	37.761	16.55	23470-00-0	330.5	$C_{19}H_{38}O_4$	Hexadecanoic acid, 2-hydroxy-1-(hydroxymethyl)ethyl ester
49.	39.230	0.58	64-17-5	697.04	$C_{41}H_{77}F_5O_2$	Octatriacontyl pentafluoropropionate
50.	40.962	11.02	621-61-4	358.55	$C_{21}H_{42}O_4$	Octadecanoic acid, 2-hydroxy-1-(hydroxymethyl)ethyl ester
